# Urgent Call to Ensure Clean Air For All in Europe, Fight Health Inequalities and Oppose Delays in Action

**DOI:** 10.3389/ijph.2024.1606958

**Published:** 2024-02-01

**Authors:** Ebba Malmqvist, Zorana Jovanovic Andersen, Joseph Spadaro, Mark Nieuwenhuijsen, Klea Katsouyanni, Bertil Forsberg, Francesco Forastiere, Barbara Hoffmann

**Affiliations:** ^1^ Division of Occupational and Environmental Medicine, Faculty of Medicine, Lund University, Lund, Sweden; ^2^ Department of Public Health, Faculty of Health and Medical Sciences, University of Copenhagen, Copenhagen, Denmark; ^3^ Spadaro Environment Research Consultants (SERC), Philadelphia, PA, United States; ^4^ Barcelona Institute for Global Health (ISGlobal), Barcelona, Spain; ^5^ Universitat Pompeu Fabra (UPF), Barcelona, Spain; ^6^ CIBER Epidemiología y Salud Pública (CIBERESP), Madrid, Spain; ^7^ Imperial College, London, United Kingdom; ^8^ Department of Public Health and Clinical Medicine, Umeå University, Umeå, Sweden; ^9^ Institute for Occupational, Social and Environmental Medicine, Centre for Health and Society, Medical Faculty, University of Düsseldorf, Düsseldorf, Germany

**Keywords:** air pollution, health, air quality, legislation, European Commission

## Introduction

As part of the Green Deal, the European Union’s (EU) ambitious plan to be the first climate neutral continent by 2050, EU launched the Zero Pollution Action Plan in 2021. One of the key elements of this plan is an update of the current air quality legislation, the EU Ambient Air Quality Directive (AAQD) with air pollution limit values of 25 μg/m^3^ for particulate matter with diameter <2.5 µm (PM_2.5_) and 40 μg/m^3^ for nitrogen dioxide (NO_2_). The need for a revision became clearer upon release of the World Health Organization Air Quality Guidelines (WHO AQG) in 2021, which recommends limit values (annual mean) of 5 μg/m^3^ for PM_2.5_ and 10 μg/m^3^ for NO_2_, based on a comprehensive global review of the key scientific evidence on health effects of ambient air pollution [1]. The difference between these values exposes the large gap between science-based standards aimed at protecting health and the current, outdated EU AAQD.

The health community position is clear: follow the science and fully align the new EU limit values with the WHO AQD by 2030. This is a historic opportunity towards clean air in Europe for all that could prevent hundreds of thousands of premature deaths and millions of new cases of non-communicable diseases (NCDs) every year [2], as well as improve health of all European citizens. Full alignment with WHO AQG would enhance children health in Europe by improving lung function [3] and reducing asthma and respiratory infections burden. Achieving the WHO AQG would also reduce healthcare costs, social, environmental and health inequalities, boost economic growth, and help mitigate the adverse effects of climate change [4].

In October 2022 the European Commission presented its proposal for a revised AAQD with limit values of 10 ug/m^3^ for PM_2.5_ and 20 μg/m^3^ for NO_2_ to be met by 2030. While the Commission’s proposal is an important step in the right direction, it provided no clear pathway on full alignement with WHO AQG [5]. In September 2023, the European Parliament went a step further, and voted to adopt the WHO AQGs with full implementation by 2035. Parliament’s historical vote to endorse science-based air quality standards was applauded for by the health community. However, in November 2023, the European Council adopted its negotiating mandate (the Council version of the AAQD proposal), which endorsed the Commission proposal, leaving out full alignment with WHO AQGs. Furthermore, the Council proposed another serious relaxation of the AAQD ambition, allowing delays in achieving limit values up to the 2040 for countries whose gross domestic product (GDP) *per capita* is below EU average, 17 of 27 EU countries, mainly in Eastern Europe and Italy [6]. The delay in cleaning up air pollution would widen the social, economic and health inequality between East and West. The Council’s proposal also allows for a ten-year delay for all countries that could demonstrate that they will not reach the limit values by 2030.

Currently (January 2024), the European Council, Parliament, and Commission are engaged in trilateral discussions of the revision of the AAQD. The lack of pathway to full alignment with WHO AQGs and potential delays are of great concern to the health professional’s community, patients, and general public.

## Delays Mean Lost Lives and Poor Health

PM_2.5_ caused 432,000 premature deaths in Europe in 2021, of which 253,000 were at levels above the recommended WHO AQG of 5 μg/m^3^ [2]. These numbers are likely underestimated, as newest research from Europe points at even stronger impacts of air pollution on mortality. Furthermore, there are millions of air pollution-related new cases of asthma, chronic obstructive respiratory diseases (COPD), acute respiratory infections, lung cancer, stroke, myocardial infarction, hypertension, diabetes, dementia and mental health disorders, as well as aggravations of these diseases in already ill persons each year.

**FIGURE 1:**
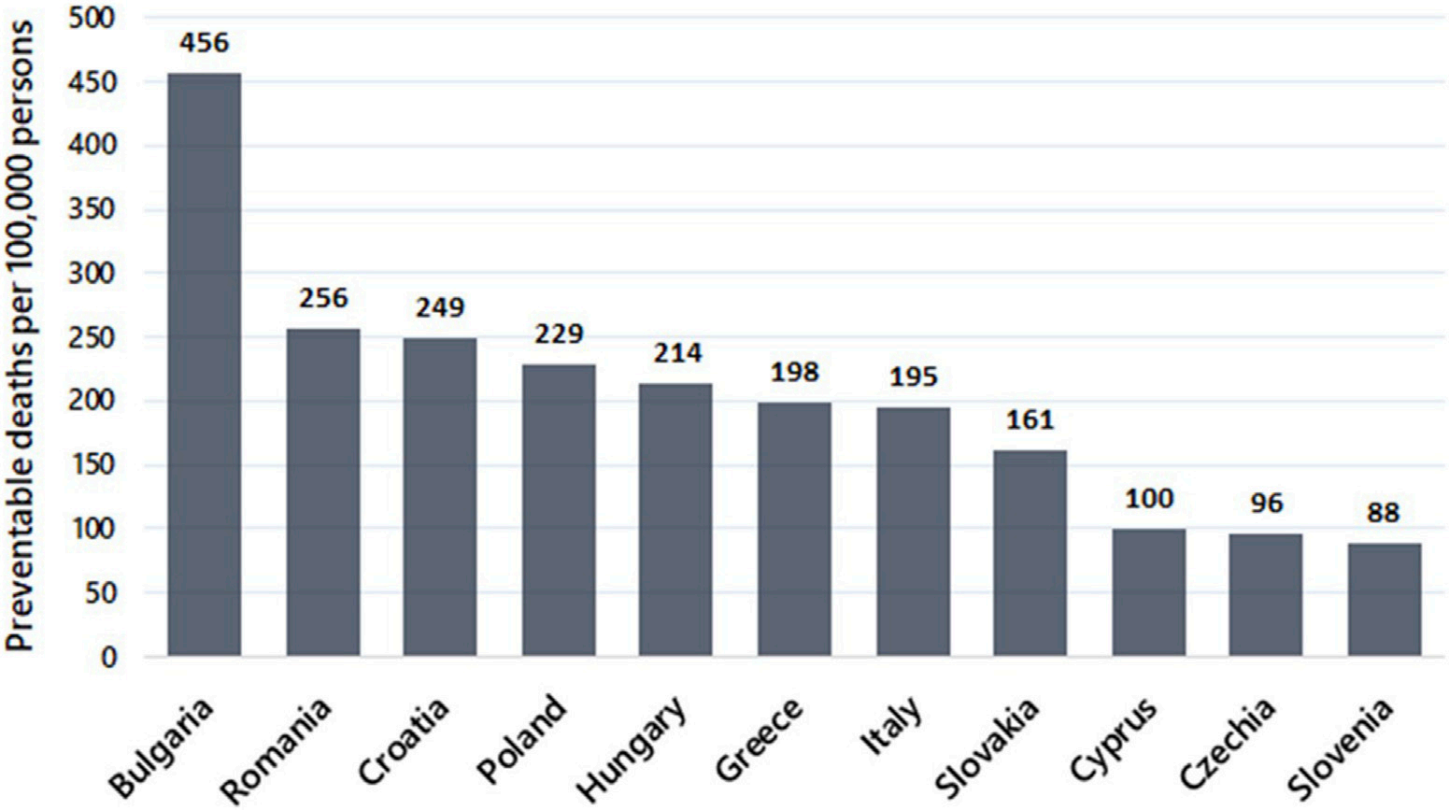
Number of preventable premature deaths (per 100,000 persons) for avoiding a 10-year delay in reaching a PM_2.5_ target concentration of 10 μg/m^3^ in Member States with a mean population-weighted PM_2.5_ exposure above 10 μg/m^3^ in 2020.

Delays in cleaning up the air in Europe are highly problematic, as they would result in preventable loss of life and exacerbate inequities across Europe. For example, for EU Member States with a mean population-weighted PM_2.5_ exposure above 10 μg/m^3^ in 2020 [7], a 10-year delay in reaching 10 μg/m^3^ (i.e. 2040 instead of 2030) would result in an excess of 327,600 premature deaths. This calculation assumes a linear decrease of PM_2.5_ levels from 2020 to 10 μg/m^3^ in either 2030 or 2040 and uses the relative risk estimate from the meta-analyses on PM_2.5_ and all-cause mortality WHO [8]. It is notable that two-thirds of the preventable health burden affects the poorer countries in Eastern Europe, and about one-third in Italy (Figure 1). These numbers make it clear that allowing delays will impose a substantial, unjust, and unacceptable loss of human lives in Europe. It is important to stress that delays will mean failure to protect those who are most susceptible to harmful effects of air pollution: children, pregnant women, elderly, already sick, and people in low socio-economic groups.

## Delays Will Widen the Inequality Gap Between East and West Europe

The proposed delay by the Council would mean that by 2030, residents of the 11 most affluent EU states could legally demand to be protected from the dangers of air pollution, while over 240 million people in 17 lower-income EU countries would still be exposed to harmful air pollution for another 10 years [6]. Eastern European countries are those that have the highest levels of air pollution and related health costs in Europe. The emissions come from coal-based energy production and outdated industry sectors, followed by use of wood and coal for residential heating and cooking, and old vehicle fleets [9]. Council proposal would increase these inequalities. Using poverty as an excuse to fail to act, is the exact opposite of what these countries need. It would be more beneficial for health with a fair and clear legislative framework and financial support targeted to accelerate (and not delay) urgently needed clean air actions and policies in all relevant sectors. This would allow the EU citizens from the most affected countries to catch up with in reaching clean air targets and enjoy health benefits as citizens of Western Europe. There are currently EUR 147 billion, or 8.3% of the multiannual financial framework for the 2021–2027 period of the EU budget dedicated to the clean air objective [10].

## Delays on Urgent Action on Air Pollution Mean Delays in Additional Health Benefits

As the healthcare systems around Europe struggle with increasing costs related to ageing and multi-morbidities, saving costs by preventing air pollution related NCDs and enhancing healthy ageing, is an opportunity that must not be missed. Finally, strict air pollution policies and initiatives would provide additional health co-benefits by enhancing physical activity (e.g., supporting a shift to active travel such as cycling, walking, and public transport in cities), reducing road traffic noise, increasing greening of the cities, and help mitigate climate change impacts on our societies and our health.

## Conclusion

Trialogue negotiations between the EU Council, Commission and Parliament have started. A deal must soon be reached. We strongly urge the EU environment ministers to put European health and environmental justice at the core of their political aspirations. This is a unique public health opportunity for EU Member States to follow the scientific evidence and listen to the concerns of citizens.
